# Structural basis of unisite catalysis of bacterial F_0_F_1_-ATPase

**DOI:** 10.1093/pnasnexus/pgac116

**Published:** 2022-07-11

**Authors:** Atsuki Nakano, Jun-ichi Kishikawa, Atsuko Nakanishi, Kaoru Mitsuoka, Ken Yokoyama

**Affiliations:** Department of Molecular Biosciences, Kyoto Sangyo University, Kamigamo-Motoyama, Kita-ku, Kyoto 603-8555, Japan; Department of Molecular Biosciences, Kyoto Sangyo University, Kamigamo-Motoyama, Kita-ku, Kyoto 603-8555, Japan; Institute for Protein Research, Osaka University, 3-2 Yamadaoka, Suita, Osaka 565-0871, Japan; Department of Molecular Biosciences, Kyoto Sangyo University, Kamigamo-Motoyama, Kita-ku, Kyoto 603-8555, Japan; Research Center for Ultra-High Voltage Electron Microscopy, Osaka University, 7-1 Mihogaoka, Ibaraki, Osaka 567-0047, Japan; Research Center for Ultra-High Voltage Electron Microscopy, Osaka University, 7-1 Mihogaoka, Ibaraki, Osaka 567-0047, Japan; Department of Molecular Biosciences, Kyoto Sangyo University, Kamigamo-Motoyama, Kita-ku, Kyoto 603-8555, Japan

## Abstract

Adenosine triphosphate (ATP) synthases (F_0_F_1_-ATPases) are crucial for all aerobic organisms. F_1_, a water-soluble domain, can catalyze both the synthesis and hydrolysis of ATP with the rotation of the central *γε* rotor inside a cylinder made of *α*_3_*β*_3_ in three different conformations (referred to as *β*_E_, *β*_TP_, and *β*_DP_). In this study, we determined multiple cryo-electron microscopy structures of bacterial F_0_F_1_ exposed to different reaction conditions. The structures of nucleotide-depleted F_0_F_1_ indicate that the ε subunit directly forces *β*_TP_ to adopt a closed form independent of the nucleotide binding to *β*_TP_. The structure of F_0_F_1_ under conditions that permit only a single catalytic *β* subunit per enzyme to bind ATP is referred to as unisite catalysis and reveals that ATP hydrolysis unexpectedly occurs on *β*_TP_ instead of *β*_DP_, where ATP hydrolysis proceeds in the steady-state catalysis of F_0_F_1_. This indicates that the unisite catalysis of bacterial F_0_F_1_ significantly differs from the kinetics of steady-state turnover with continuous rotation of the shaft.

Significance Statement The F_0_F_1_-ATPase rotates its central axis by continuously changing the structure of the three *β* subunits upon ATP hydrolysis. Here, we reconstructed cryo-electron microscopy structures under unisite conditions that permit only a single catalytic *β* subunit per enzyme to bind ATP. The structures indicated that hydrolysis of the first ATP occurs at *β*_TP_ instead of *β*_DP_, where ATP hydrolysis proceeds in the steady-state catalysis of F_0_F_1_. This indicates that unisite catalysis is an initial reaction that is distinguished from steady-state rotary catalysis in F_0_F_1_.

## Introduction

Adenosine triphosphate (ATP) synthases (F_0_F_1_) are crucial for aerobic organisms and reside in the inner membranes of mitochondria, plasma membranes of bacteria, and thylakoid membranes of chloroplasts in plants (1–3). F_0_F_1_ consists of a hydrophilic F_1_ domain responsible for ATP hydrolysis or synthesis and a hydrophobic F_0_ domain housing proton translocation across the membranes. ATP hydrolysis/synthesis in F_1_ is coupled to proton flow in F_0_ through the rotation of a common shaft.

**Fig. 1.**
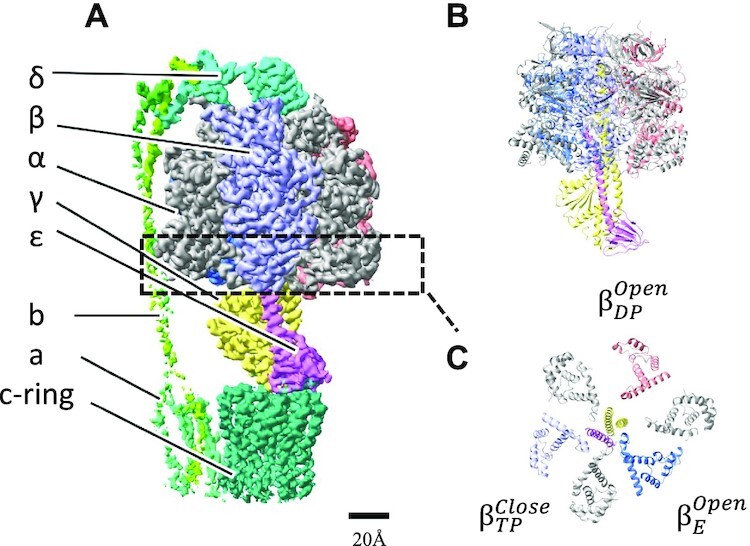
Structure of nucleotide depleted wild-type F_0_F_1_ (*ND-wt-*F_0_F_1_) of *G. stearothermophilus*. Cryo-electron microscopy density map of *ND*-*wt*-F_0_F_1_ (state 1). (A) Side view of the overall structure of F_0_F_1_ as a ribbon model. All *α* subunits are represented as gray, and other subunits are colored in F_1_ domain. (B) View of the F_0_ side of F_1_ domain at C terminal region. Superscript indicates the closed or open structure of the *β* subunit. (C) Ribbon representation of view from the F_0_ side. No nucleotide was found at noncatalytic or catalytic nucleotide-binding sites.

F_0_F_1_ from the thermophilic bacteria *Geobacillus stearothermophilus* is one of the best characterized ATP synthases because of its structural stability and simple subunit structure (*α*_3_*β*_3_*γ*_1_*ε*_1_*δ*_1_*a*_1_*b*_2_*c*_10_; Fig. 1A). In particular, single-molecule rotation experiments using this enzyme enabled direct observation of the rotation of ATP synthase, which considerably improved our understanding of the mechanochemical cycle of F_0_F_1_ (4–11).

The *γε* rotor in the F_1_ domain (*α*_3_*β*_3_*γ*_1_*δ*_1_*ε*_1_) is surrounded by a cylinder composed of three noncatalytic *α* and three catalytic *β* subunits arranged alternately (Fig. 1). The *ε* subunit modulates ATP hydrolysis activity by the structural change from a contracted to an extended form with a C-terminus helix towards *α*_3_*β*_3_ (12). Several experimental studies have demonstrated that the extended C-terminus of the ε subunit (*ε*-CT up form) strongly inhibits ATPase activity of the F_1_ domain; however, the inhibition mechanism of the *ε*-CT up form remains elusive.

In F_1_ domain, six nucleotide-binding sites are located at different interfaces between the *α* and *β* subunits; three catalytic sites are located mainly in the *β* subunit, while the other three sites, called noncatalytic nucleotide-binding sites, are located mainly in the *α* subunit. According to the binding change mechanism of ATP synthesis, the three catalytic *β* subunits in F_1_ are in different conformations (open, loose, and tight); However, they interconvert sequentially between three different conformations as catalysis proceeds (2). Thus, at a given time, all three catalytic *β* subunits are in different conformations. The crystal structure of F_1_ from bovine heart mitochondria demonstrated the following asymmetry of catalytic sites: *β*_E_, which adopts an open structure with no nucleotide; *β*_TP_, which adopts a structure containing ATP analog (AMPPNP); and *β*_DP_, which adopts a structure containing ADP (13, 14). The C-terminal region of each *β* subunit interacts with the *γ* subunit, and the differences are a consequence of the asymmetric association of the *γ* subunit with the *α*_3_*β*_3_ cylinder. Thus, sequential interconversion between three different *β* subunits drives the rotation of the *γ* subunit with ATP hydrolysis (4).

The asymmetric structures of *α*_3_*β*_3_ were found in the F_1_ structure of other species (15–17) and V-ATPases (16), which is another rotary ATPase that is evolutionarily and structurally related to F_0_F_1_. In this study, we refer to the asymmetric architecture as a “Walker structure.”

Assuming that F_0_F_1_ adopts the Walker structure during ATP hydrolysis, the alternating participation of *β* subunits within ATP hydrolysis does not require positive cooperation. Early studies showed that F_1_-ATPases exhibited multiple *K*_m_, likely due to the activation of F_1_-ATPase by the binding of ATP to the *α* subunits (18). However, using both bulk and single-molecule experiments, one rotary mechanism was found to govern the entire range of nanomolar to millimolar ATP (19). This indicates that thermophilic F_1_-ATPase obeys simple Michaelis–Menten kinetics with a single *K*_m_ value. However, previous experiments using a mitochondrial F_1_ indicated strong positive cooperation between the catalytic sites. F_1_-ATPase was compared under unisite and multisite catalytic conditions in which the ATP/enzyme ratio was adjusted to facilitate operation of either one or three sites (20). The multisite/unisite rate enhancement ratio of 10^6^ fold was interpreted as a reflection of the strong positive cooperation among the three catalytic sites. The unisite catalysis experiment also suggests a high affinity of unisite to ATP with a binding constant *K*_d_ of ∼10^−12^ M, which is markedly higher than the *K*_m_ of ∼10^–5^ M in bacterial F_1_ (19, 21). For thermophilic F_1_-ATPase, similar ATP hydrolysis in a single catalytic site has been reported (21–23). However, the structural basis of the high affinity for ATP observed in unisite catalysis and whether unisite catalysis reflects steady-state activity (multisite catalysis) remains elusive.

In this study, we constructed a mutant F_0_F_1_ with a C-terminus-truncated *ε* subunit and showed that it does not undergo *ε* inhibition. The structure of this mutant F_0_F_1_ was determined using cryo-electron microscopy (cryo-EM) and compared with that of wild-type (*wt*) F_0_F_1_ to understand the structural basis of *ε* inhibition. Furthermore, we determined the structure of the mutant F_0_F_1_ under unisite catalytic conditions to capture the structure after ATP hydrolysis of the *β* subunit.

## Results

### Cryo grid preparation of *wt*-F_0_F_1_ and ATPase-active Δ*εCT*-F_0_F_1_

In this study, we used purified F_0_F_1_ from *G. stearothermophilus* expressed in *Escherichia coli* (24). The ATPase activity of *wt-*F_0_F_1_ is ∼10 s^−1^ (Supplementary Fig. S1 and Supplementary Table S1), which is considerably lower than that of F_1_ (*α*_3_*β*_3_*γ*, ∼70 s^−1^) (25). Several studies have indicated that ATP hydrolysis by *G. stearothermophilus* F_0_F_1_ is significantly inhibited when the extended C-terminal region of the *ε* subunit penetrates the *α*_3_*β*_3_ cavity (12, 26–28). The initial ATPase activity of *wt*-F_0_F_1_ was very low because of the initial lag (Supplementary Fig. S1). To obtain ATPase-active F_0_F_1_, we constructed a mutant F_0_F_1_ with a C-terminal-truncated *ε* subunit (Δ*εCT*-F_0_F_1_). The prepared Δ*εCT*-F_0_F_1_ was subjected to dialysis in phosphate-buffered saline to deplete the bound nucleotide, as described in the *Materials and Methods* section. For the nucleotide-depleted thermophilic F_0_F_1_ (*ND-*Δ*εCT*-F_0_F_1_), the lag time was shorter than that of *wt*-F_0_F_1_, and the ATPase activity at 1,000 s after the start of reaction was 130 s^−1^ (Supplementary Table S1), which was comparable to the ATPase activity of *wt*-F_1_ without the *ε* subunit (5, 25).

First, we prepared a cryo grid of nucleotide-depleted *wt*-F_0_F_1_ (*ND-wt*-F_0_F_1_) in the absence or presence of 4 mM ATPγS. In addition, the cryo grid of Δ*εCT*-F_0_F_1_ under unisite conditions (molar ratio of enzyme to ATP is approximately 1:4.) was used for structural analysis. These cryo grids were subjected to cryo-EM image acquisition using a Titan Krios (Thermo Fisher Scientific) equipped with a K3 direct electron detector.

### Structure of *ND-wt*-F_0_F_1_

Flowcharts showing the image acquisition and reconstitution of the 3D structure of nucleotide-depleted *wt*-F_0_F_1_ (*ND-wt-*F_0_F_1_) are summarized in Supplementary Fig. S2. We reconstructed three rotational states (state 1: 3.1 Å, state 2: 3.0 Å, and state 3: 3.7 Å resolutions) from the single-particle images of *ND*-*wt*-F_0_F_1_. The structure of *ND-wt*-F_1_ domain is similar to the structure of F_1_ domain in *wt*-F_0_F_1_ reported in a study by Guo et al.(6N2Y(12)), which adopted the following structures: “open in *β*_E_ without nucleotide,” “closed in *β*_TP_ containing ADP,” and “open in *β*_DP_ without nucleotide.” In fact, the three *β* subunits in *ND-wt*-F_1_ are similar to their counterparts in *wt*-F_0_F_1_ (Supplementary Fig. S3). In the structure of *ND-wt*-F_0_F_1_, the C-terminal helix of the *ε* subunit (*ε-CT*) also adopts the up form and penetrates the cavity of the *α*_3_*β*_3_ cylinder (Fig. 1B). In contrast to the previous structure of *wt*-F_0_F_1_ (12), no nucleotide density was observed at the noncatalytic nucleotide-binding site in the three *α* subunits or the catalytic site in *β*_TP_ (Figs. 1C and 3A). Instead, a density likely corresponding to a phosphate was observed at the catalytic site of *β*_TP_ (Fig. 3A and Supplementary Fig. S4A). This phosphate was possibly derived from the phosphate buffer used for nucleotide depletion.

Therefore, the structure indicates that the closed structure of *β*_TP_ is stabilized by the penetration of *ε-CT* into the cavity between *α*_DP_ and *β*_TP_, which is independent of nucleotide binding to *β*_TP_.

### Structure of *wt*-F_0_F_1_ exposed to 4 mM ATPγS

**Fig. 2.**
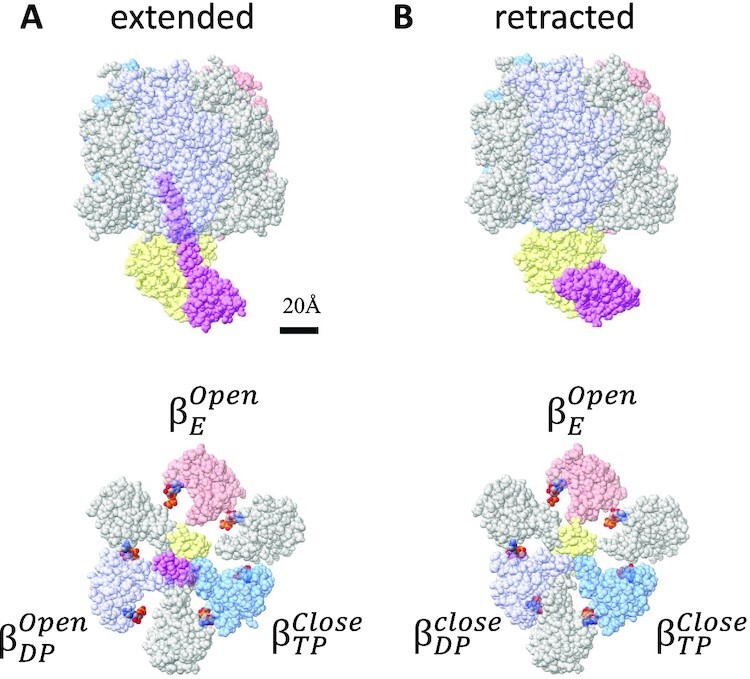
Structure of wild-type F_0_F_1_ (*wt-*F_0_F_1_) exposed to 4 mM ATPγS. Structures of *wt*-F_0_F_1_ exposed to 4 mM ATPγS with the retracted (A) or extended (B) *ε*-subunit viewed from vertical side (upper) and from F_1_ side (lower). The bound nucleotides are represented as color spheres. All *α* subunits are represented in gray, and all *β* subunits are colored.

**Fig. 3.**
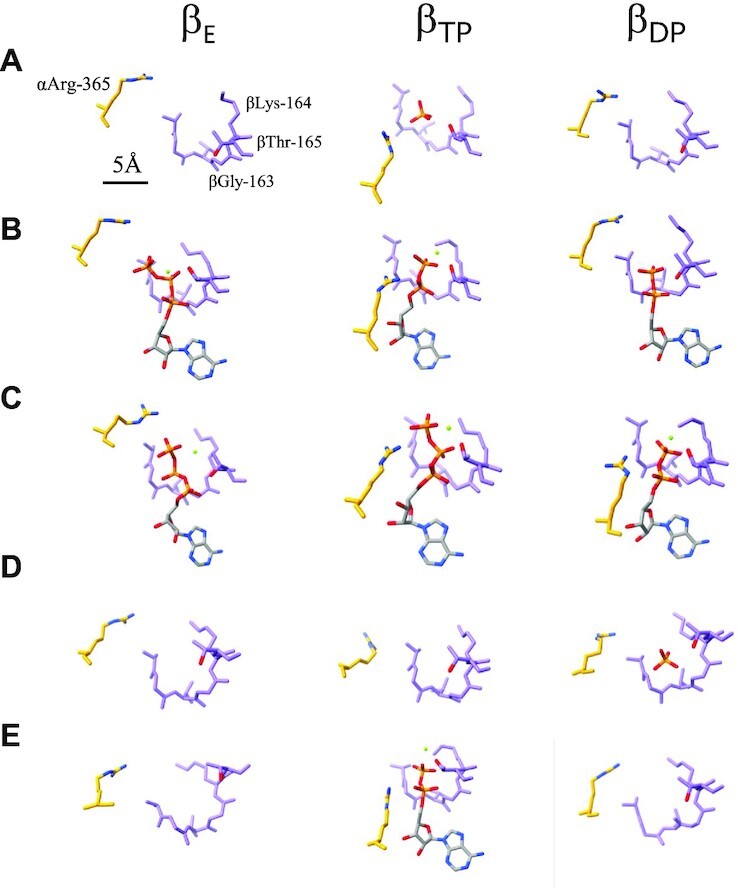
Structure of catalytic sites of wild-type and mutated F_0_F_1_ under different condition. Magnified views of the three catalytic sites (*β*_E_, *β*_TP_, and *β*_DP_) in each structure are shown as follows: (A) *ND-wt*-F_0_F_1_, (B) *wt*-F_0_F_1_*ε* up form with ATPγS, (C) *wt*-F_0_F_1_*ε* down form with ATPγS, (D) *ND-ΔCT*-F_0_F_1_, and (E) *US-ΔCT*-F_0_F_1_. Bound nucleotides and Mg ions are highlighted as stick and spherical representations, respectively. Scale bar is 5 Å.

Previous studies have indicated that the binding of ATP to an isolated *ε* subunit induces a conformational change in *ε-CT* from the “up” to “down” form (25, 29). The ATPase activity of *wt*-F_0_F_1_ gradually accelerated in the presence of 4 mM ATP (Supplementary Fig. S1 and Supplementary Table S1). In this study, we determined the cryo-EM structure of F_0_F_1_ exposed to 4 mM ATPγS, which is a slow hydrolyzable ATP analog (30), to prove the conformational change of the *ε* subunit in the complex. After focused 3D classification using a mask covering the F_1_ domain (Supplementary Fig. S5B), we obtained the following two structures: F_1_ with the up form of *ε* subunit from 526,524 particles at 2.6 Å resolution and F_1_ with the down form of *ε* subunit from 39,991 particles at 3.3 Å resolution (Fig. 2 and Supplementary Fig. S5B). In the down form of *ε* subunit in the complex, the density of bound ATPγS was identified with similar coordination of surrounding amino acid residues to that in the monomeric *ε* subunit (Supplementary Fig. S6) (31). F_0_F_1_ with the down form of *ε* subunit adopted the following canonical Walker structures: “open in *β*_E_ with ATPγS,” “closed in *β*_TP_ with ATPγS,” and “closed in *β*_DP_ with ADP ” (Figs. 2B and 3C). ATPγS molecules were also identified at the noncatalytic sites of the three *α* subunits (Fig. 2, lower). In contrast, the structure of F_0_F_1_ with the extended *ε-CT* was similar to the *ND-wt*-F_0_F_1_ structure, except the nucleotide occupancy of “open in *β*_E_ with ATPγS,” “closed in *β*_TP_ with ADP,” and “open in *β*_DP_ with ADP” (Fig. 3B). These results indicate that the extended *ε-CT* hampers the conformational change of the F_1_ domain from the inhibitory conformation with “open in *β*_E_,” “closed in *β*_TP_,” and “open in *β*_DP_” to the Walker structure with “open in *β*_E_,” “closed in *β*_TP_,” and “closed in *β*_DP_” during ATP hydrolysis.

### Structure of Δ*εCT*-F_0_F_1_ under unisite catalysis conditions

To capture the structure of the unisite catalysis of F_0_F_1_, 15 µM of *ND-*Δ*εCT*-F_0_F_1_ was mixed with 4 µM of the ATP-containing regeneration system described in the *Materials and Methods* section. The mixture was incubated for 120 s at 25°C, then loaded onto a holey grid, and subjected to flash freezing.

We obtained multiple F_0_F_1_ structures with and without nucleotides (*ND-*Δ*εCT*-F_0_F_1_) in the *β*_TP_ using 418,497 selected particle images of Δ*εCT*-F_0_F_1_ (Supplementary Fig. S7B). For *ND-*Δ*εCT*-F_0_F_1_, the structures of two rotational states without nucleotides were obtained at the following resolutions: state 1 at 3.6 Å and state 2 at 3.4 Å, with the *γ* subunit positions differing by 120° in each state. The structure of state 3 without nucleotides was not identified because of the small number of particles. Further, we obtained the F_1_ parts of states 1 and 2 at resolutions of 3.4 Å and 3.3 Å, respectively, using focused refinement of the F_1_ domain (Supplementary Fig. S7B).

**Fig. 4.**
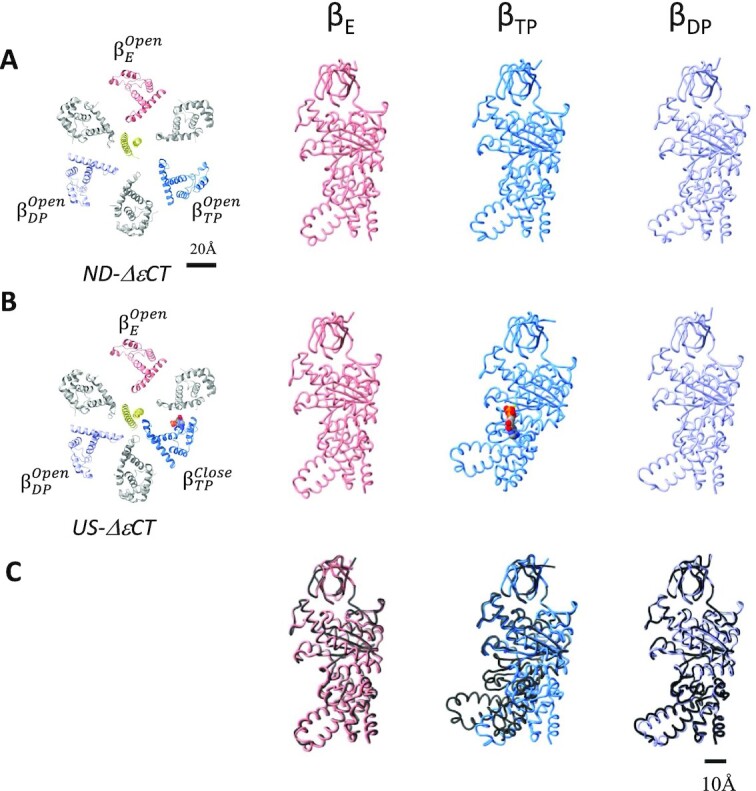
Comparison of the structure of nucleotide-depleted *ΔεCT*-F_0_F_1_ with that of *US*-*ΔεCT*-F_0_F_1_. Cryo-electron microscopy structures of *ND-ΔεCT*-F_0_F_1_ (A) and *ΔεCT*-F_0_F_1_ under unisite conditions (B). The slice view of the F_1_ domain is represented in the left panel, and the main chain of each *β* subunit is lined. *Right panels*, each *β* subunits (*β*_TP_, *β*_DP_, and *β*_E_) of *ND*-*ΔεCT*-F_0_F_1_ or *US*-*ΔεCT*-F_0_F_1_ is represented as a colored chain. (C) Comparison of the *β* subunits between *ND*-*ΔεCT*-F_0_F_1_ and *US*-*ΔεCT*-F_0_F_1_. Each *β* subunit (*β*_TP_, *β*_DP_, and *β*_E_) of *ND*-*ΔεCT*-F_0_F_1_(colored chain) or *US-ΔεCT*-F_0_F_1_ (black chain) is superimposed on the *β*-barrel domain (1 to 80 amino acids).

The structure of *ND-*Δ*εCT*-F_0_F_1_ significantly differs from the structure of *ND-wt*-F_0_F_1_ in which three catalytic *β* subunits adopt “open in *β*_E_,” “closed in *β*_TP_,” and “open in *β*_DP_” (Fig. 1C). In contrast, all three *β* subunits in *ND-*Δ*εCT*-F_0_F_1_ adopt an almost identical structure of open conformations as follows: “open in *β*_E_,” “open in *β*_TP_,” and “open in *β*_DP_” (Fig. 4A). There was no nucleotide bound to the three open *β* subunits of *ND-*Δ*εCT*-F_0_F_1_, but a density corresponding to phosphate was observed in the *β*_DP_ (Fig. 3D and Supplementary Fig. S4D). This phosphate may be derived from the phosphate buffer used for nucleotide depletion. Our findings suggest that nucleotide depletion from the F_1_ domain causes all three *β* subunits to adopt an open conformation. The interaction of the *γ* subunit with the three open *β* subunits are shown in Supplementary Fig. S8. The C termini region of closed *β*_TP_ is in close proximity to the coiled coil of the *γ* subunit, while the open *β*_TP_ is in close proximity to the globular domain of the *γ* subunit (Supplementary Fig. S8C).

In other words, the penetration of extended *ε-CT* into the *α*_3_*β*_3_ cavity does not result in the open conformation of *β*_DP_ but rather forces open *β*_TP_ without bound nucleotides to the closed conformation (cf. Figs. 1C and 4A).

For *ND-*Δ*εCT*-F_0_F_1_ containing a nucleotide in *β*_TP_, we determined the structures of the following three states: state 1 at 3.2 Å, state 2 at 3.4 Å, and state 3 at 4.0 Å. For state 1, another subclass with nearly identical structure (state 1) was isolated (Supplementary Fig. S6C). Upon focused refinement using an F_1_ mask, the structure of the F_1_ domain for each state was obtained (Supplementary Fig. S6B, lower). We refer to the Δ*εCT*-F_0_F_1_ structure containing a nucleotide at the *β*_TP_ as UniSite*-*Δ*εCT*-F_0_F_1_ (*US-*Δ*εCT*-F_0_F_1_). The structure of *US-*Δ*εCT*-F_0_F_1_ was different from that of *ND-*Δ*εCT*-F_0_F_1_. The structures of two *β* subunits, *β*_E_ and *β*_DP_, adopted an open form without nucleotides, whereas *β*_TP_ adopted a closed form containing nucleotide density (Fig. 3E). These findings indicate that the structure of *US-*Δ*εCT*-F_0_F_1_ is very similar to that of *ND-wt*-F_0_F_1_. Specifically, F_0_F_1_ in both structures adopt “open in *β*_E_,” “closed in *β*_TP_,” and “open in *β*_DP_.” In addition, the relative position of the *γ* subunit to *α*_3_*β*_3_ was completely analogous in the two structures (Supplementary Fig. S9). The structure of *US-*Δ*εCT*-F_0_F_1_ revealed a nucleotide density due to ADP at the catalytic site in *β*_TP_, indicating that ATP bound to *β*_TP_ was already hydrolyzed (Figs. 3E and 4B).

Furthermore, the position of the *γ* subunit of *US-*Δ*εCT*-F_0_F_1_ relative to *α*_3_*β*_3_ is slightly different from that of *ND-*Δ*εCT*-F_0_F_1_. Superimposition of the two structures with *α*_3_*β*_3_ shows ∼ 7° rotation of the *γ* subunit in the hydrolysis direction (Supplementary Fig. S8A). Assuming that the first ATP binds to *β*_TP_, the rotation of the *γ* subunit driven by unisite catalysis is faint compared to the 120° rotation of the *γ* subunit upon binding and hydrolysis of one ATP molecule under ATP-saturated conditions (multisite conditions).

## Discussion

In this study, we reveal that substoichiometric ATP is hydrolyzed at the *β*_TP_ of *ND-*Δ*εCT*-F_0_F_1_ under unisite catalysis conditions, where a single catalytic site per enzyme molecule binds ATP.

**Fig. 5.**
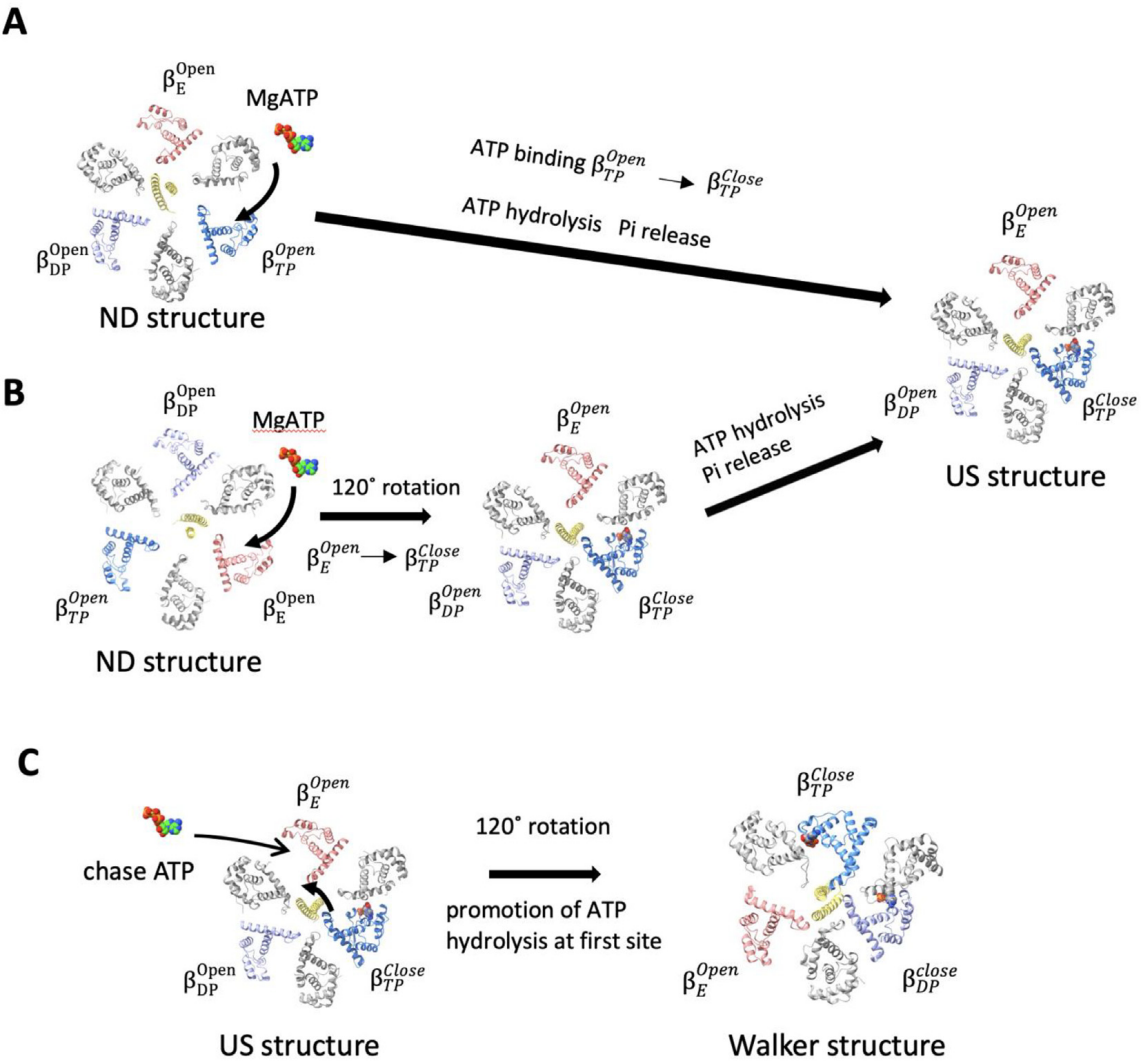
Schematic representation of possible catalytic pathways for unisite catalysis at *β*_TP_ and promotion of ATP hydrolysis by chase ATP. Three *β* subunits (*β*_TP_, *β*_DP_, and *β*_E_) and *γ* subunits are colored in blue, blue–purple, magenta and yellow, respectively. All *α* subunits are represented as gray. (A) First ATP binds to *β*_TP_ in the nucleotide-depleted (*ND*) structure where the three *β* subunits are open. Then, $\beta _{TP}^{Open}$ changes to $\beta _{TP}^{close}$ with hydrolysis of bound ATP. The conformation change of *β*_TP_ induces a 7° rotation of the *γ* subunit. The phosphate is released spontaneously, resulting in the unisite structure (*US*). (B) The first ATP binds to *β*_E_ in the *ND* structure, followed by structural changes of three *β* subunits with 120° rotation of the *γ* subunit. (C) The bound ATP on *β*_TP_ is slowly hydrolyzed by coordination of catalytic amino acid residues not suitable for hydrolysis of ATP. Binding of the second ATP to *β*_E_ causes 120° rotation of *γ* subunit and structural transition of the each of the following *β* subunits: *β*_E_ to *β*_TP_, *β*_TP_ to *β*_DP_, and *β*_DP_ to *β*_E_. The *β* subunit to which the first ATP binds becomes *β*_DP_, and the hydrolysis of ATP is accelerated.

All three *β* subunits in *ND-*Δ*εCT*-F_0_F_1_ adopted an open form, implying that the first ATP can bind to any of the *β* subunits. Assuming that the first ATP binds to *β*_TP_, the conformation change from the open to the closed form of *β*_TP_ occurs without the 120° rotation of *γ* subunit (Fig. 5A), which is consistent with previous studies suggesting unisite catalysis without rotation of *γ* subunit in *E. coli* F_1_ (32). In the *wt*-F_0_F_1_ structure, ADP is bound only to *β*_TP_, and both *β*_DP_ and *β*_E_ are in the open form without nucleotides, which suggests that *β*_TP_ can easily change to the closed form upon ATP binding, particularly when compared to *β*_DP_ and *β*_E_. *β*_TP_ bound with ATP immediately changes to the closed form due to the zippering motion by the bound ATP, which likely increases the affinity of *β*_TP_ for ATP. This is consistent with the high affinity for ATP reported in unisite catalysis experiments (20, 22, 33). Alternatively, it is also possible that the first ATP bound to *β*_E_ and the *γ* subunit rotated 120° with a structural change of the ATP-bound *β*_E_ to *β*_TP_, resulting in the structure of *US-*Δ*εCT*-F_0_F_1_. Although both catalytic pathways (Fig. 5A and B) exhibit high possibilities for unisite catalysis, it is more likely that the first ATP binds to *β*_TP_ to explain the high affinity of the unisite for ATP.

Both *β*_DP_ and *β*_E_ retain open structures after unisite catalysis at *β*_TP_; therefore, the next catalytic event is ATP binding to either *β*_DP_ or *β*_E_. During ATP hydrolysis by F_0_F_1_, each conformational change from *β*_E_ to *β*_TP_, *β*_TP_ to *β*_DP_, and *β*_DP_ to *β*_E_ occurs with 120° rotation of the *γ* subunit. Assuming that ATP then binds to *β*_E_ and induces a conformational change of each *β* subunit with 120° rotation of the *γ* subunit, F_0_F_1_ adopts the following canonical Walker structure observed in most F_1_ structures: “open in *β*_E_,” “closed in *β*_TP_ with the *second* ATP,” and “closed in *β*_DP_ with the ADP from *first* ATP” (Fig. 5C). Several studies using various F_1_-ATPases have reported that hydrolysis of ATP bound to the first catalytic site is markedly accelerated by the addition of an excess ATP (cold chase experiment) (20, 21, 35). In Walker structures, *β*_TP_ and *β*_DP_ adopt very similar closed conformations; however, their catalytic sites are not equivalent (Supplementary Fig. S10). Although these differences are relatively small, they have a marked effect on catalysis. ATP in *β*_DP_ is immediately hydrolyzed, whereas hydrolysis of ATP in *β*_TP_ proceeds slowly (14, 33). Therefore, most crystal structures of F_1_ contain ATP analogs in *β*_TP_ and ADP in *β*_DP_. Upon binding of ATP to *β*_E_ by the addition of excess ATP, the conformational change of *β*_TP_ to *β*_DP_ promoted the hydrolysis of ATP at the catalytic site (Fig. 5C).

The F_0_F_1_ structures exposed to 4 mM ATPγS provided direct evidence of conformational changes in the *ε* subunit by ATP binding in the holo-F_0_F_1_ complex. The retracted ε subunit in the complex contains ATP with the similar coordination as the monomeric *ε* subunit (Supplementary Fig. S5) (31). In another *wt*-F_0_F_1_ structure exposed to 4 mM ATPγS concentration, the *ε*-inhibited structure, which adopted “open in *β*_E_,” “closed in *β*_TP_,” and “open in β_DP_,” was maintained, although all nucleotide binding sites were occupied with ADP or ATPγS (Figs. 2 and 3B). This indicates that the inhibited *wt*-F_0_F_1_ structure by the extended *ε* subunit is not activated by ATP binding to *β*_E_ or *β*_DP_ (Fig. 2A), and that the conformational change of the *ε* subunit by ATP binding is crucial for the activation of *wt*-F_0_F_1_ for ATP hydrolysis activity (Fig. 2B).

In this study, we determined the multiple structures of F_0_F_1_ ATPase during catalysis by structural analysis using cryo-EM, which allows the capture of states inaccessible to crystallization. The techniques and approaches used in this study can potentially assist the elucidation of the detailed reaction and regulatory mechanisms of other enzymes.

## Materials and methods

### Protein purification

*Wt*-F_0_F_1_-ATP synthase from *G. stearothermophilus* was purified from *E. coli* DK 8 strain containing an expression vector (pTR19-ASDS) for F_0_F_1_, as described previously (24). The expression vector for *ΔεCT*-F_0_F_1_, which lacks the C-terminal of *ε* subunit (83 to 133 amino acids), was constructed from pTR19-ASDS. Transformed *E. coli* cells were grown in 2 × YT medium at 37°C for 16 h before harvesting by centrifugation at 5000 × *g*. The cell pellet was suspended in lysis buffer (50 mM Tris–Cl pH 8.0, 5 mM MgCl_2_, and 10% [w/v] glycerol), and the cell membranes were collected by ultracentrifugation at 35,000 rpm for 20 min and solubilized by mixing in solubilization buffer (50 mM Tris–Cl, 5 mM MgCl_2_, 10% [w/v] glycerol, and 2% n-dodecyl-d-maltoside [DDM]) at 4°C for 3 h. The supernatant was then applied to a Ni-NTA column. For bound nucleotide removal, the eluted fractions containing F_0_F_1_ were dialyzed against 200 mM sodium phosphate (pH 8.0), 10 mM EDTA, and 0.03% DDM overnight at 25°C and the dialysis buffer was changed thrice. The dialyzed F_0_F_1_ fractions were concentrated using ultrafiltration with an Amicon filter (100 KDa cut-off, Amicon corp.) and loaded onto a Superose 6 Increase 10/300 column (Cytiva) equilibrated with gel permeation buffer (20 mM Tris–HCl pH 8.0, 150 mM NaCl, and 0.03% DDM). The peak fractions (6 to 9 mg/mL) were used for the cryo grid preparation or ATPase assay.

### Grid preparation

For cryo grid preparation, Quanfifoil R1.2/1.3 Mo grids were glow-discharged for 1 min using an Ion Bombarder (Vacuum Device). Prior to blotting, 3 µL of the samples were placed on a cryo grid and incubated for 15 min. Further, 2.6 to 3.5 µL F_0_F_1_ was loaded onto the grid and blotted for 3 to 10 s with a blot force of 10, drain time of 0.5 s, and 100% humidity using a FEI Vitrobot (ThermoFisher). The blotted grid was then plunged into a liquid ethane. For the unisite catalysis condition, 1 µL reaction buffer (0.2 M Tris–Cl pH 8.0, 40 µM ATP, 40 mM PEP, 1 M KCl, and 5 mg/mL pyruvate kinase) was added to 9 µL of the sample. The mixtures were incubated for 60 s at 25°C, followed by blotting and vitrification.

Cryo-EM imaging under ATPγS and *US* conditions was performed using a Titan Krios (FEI/Thermo Fisher Scientific) operating at 300 kV acceleration voltage and equipped with an electron detector K3 (Gatan) in electron counting mode (CDS). Cryo-EM imaging under *wt*-*ND* conditions was performed in CDS using CRYOARM 300 (JEOL) operating at 300 kV and an electron detector K3 (Gatan).

Data collection was performed using SerialEM software with a calibrated magnification of 0.88 Å pixel^–1^ for the ATPγS and *ΔεCT-US* conditions and a calibrated magnification of 1.01 Å pixel^–1^ for the *wt*-*ND* condition. Under *wt*-*ND* and ATPγS conditions, data were collected at an electron dose of 50.0 e^–^/Å^2^ for an exposure time of 5 s, and under unisite conditions, data were collected at an electron dose of 60.0 e^–^/Å^2^ for an exposure time of 6 s. The defocus range was 0.8 to 2.0 µm, and data were collected at 50 frames for the *wt*-*ND* condition, 55 frames for the ATPγS condition, and 59 frames for the unisite condition.

### Image processing

The details of the image-processing procedure for each condition are described in Supplementary Figs. 2, 4, and 6. Image analysis was performed using RELION 4.0β (36) and cryoSPARC v3.2 (37). The file format conversion between RELION and cryoSPARC was performed using the script csparc2star.py in Pyem. The beam-induced drift was corrected using MotionCor2 (38), and the CTF was estimated using CTFFIND 4.1 (39). We analyzed 7,329 movies for the unisite condition, 9,625 movies for the *wt*-*ND* condition, and 17,261 movies for the ATPγS condition. Particle picking was performed using Topaz (40). Good particles were selected by 2D classification and trained using 4,000 particles. Autopicking using the trained topaz model yielded 499,788 particles for the unisite condition, 1,381,269 particles for the *wt*-*ND* condition, and 1,020,321 particles for the ATPγS condition. Particles picked by Topaz were subjected to 2D classification for further selection of good particles. Then heterogeneous refinements using cryoSPARC were performed to eliminate junk particles. Further, we selected 418,497 particles for unisite conditions, 622,109 particles for *wt*-*ND* conditions, and 912,931 particles for ATPγS conditions. Heterogeneous refinement was then used to classify F_0_F_1_ into multiple conformational states. These particles were re-extracted at full pixel size and subjected to repeated 3D auto-refinement, CTF refinement, and Bayesian polishing (36). The structures of the three rotational states, including their subclasses, were obtained at 3 to 4.5 Å resolution. All obtained classes of F_0_F_1_ were subjected to focused refinement on the F_1_ part to reduce the resolution loss due to the relative motion of the F_0_ part with F_1_. In the analysis of the ATPγS dataset to determine a small percentage of *ε*-retracted structures, the class of F_0_F_1_ of all states were added together to increase the number of particles, and focused refinement was performed on the F_1_ part. The obtained F_1_ structure was subjected to focused 3D classification by masking with *γε*-*β*_DP_ to detect structural changes between the *ε* and *β* subunits. The resolution was estimated using the gold standard Fourier shell correlation (FSC) = 0.143 criterion.

### Model building and refinement

We used Phenix real-space refinement (41), ISOLDE (42), and COOT (41) for the atomic model building. The epsilon-extended F_1_ model was built using PDB 6N2Y as the initial model, and the epsilon-retracted subunit was built using PDB 2E5Y. The initial model, which was a rigid body fitted to the density map by UCSF ChimeraX (43), was first refined by Phenix real-space refinement. Residues that did not fit correctly into the map were manually placed using the COOT and ISOLDE. Refinement and manual modification were repeated until the model parameters were improved. Lastly, the refinement model was evaluated using MolProbity (44) and EMRinger (45).

## Supplementary Material

pgac116_Supplemental_File

## Data Availability

Data is available in the manuscript and supplementary materials. Cryo-EM density maps (.mrc files) and atomic models (.pdb files) obtained in this study were deposited to EMDB and PDB. The accession codes (PDBID and EMDBID) 7XKH, 7XKQ, 7XKR, 7XKO, 7XKP, 33,251, 33,252, 33,253, 33,264, 33,265, 33,266, 33,267, 33,268, 33,277, 33,258, 33,278, 33,259, 33,279, 33,260, 33,280, 33,261, 33,281, 33,269, 33,282, 33,262, 33,283, and 33,263 are summarized in Supplementary Tables S2–S5. The data that support the findings of this study are available from PDB and EMDB.
