# Supplementary material for: Structural basis of unisite catalysis of bacterial F0F1-ATPase
Source: PNAS Nexus. 2022 Jul 11;1(3):pgac116. doi: 10.1093/pnasnexus/pgac116 (PMC9896953; doi:10.1093/pnasnexus/pgac116)
Supplement: pgac116_Supplemental_File [file pgac116_supplemental_file.pdf]

Fig. S1

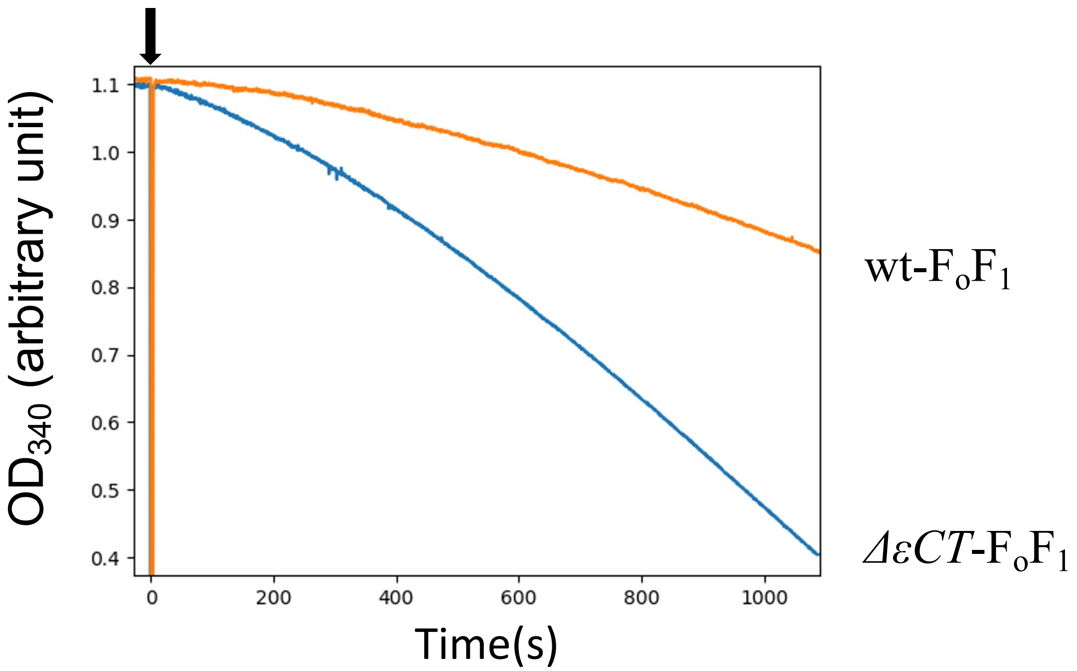

Figure S1. ATPase activity of wild-type F_oF_1 and *ΔεCT*-F_oF_1
The activities of wild-type F_oF_1 and *ΔεCT*-F_oF_1 were measured using the enzyme coupling assay described in the Materials and Methods section.

Fig. S2

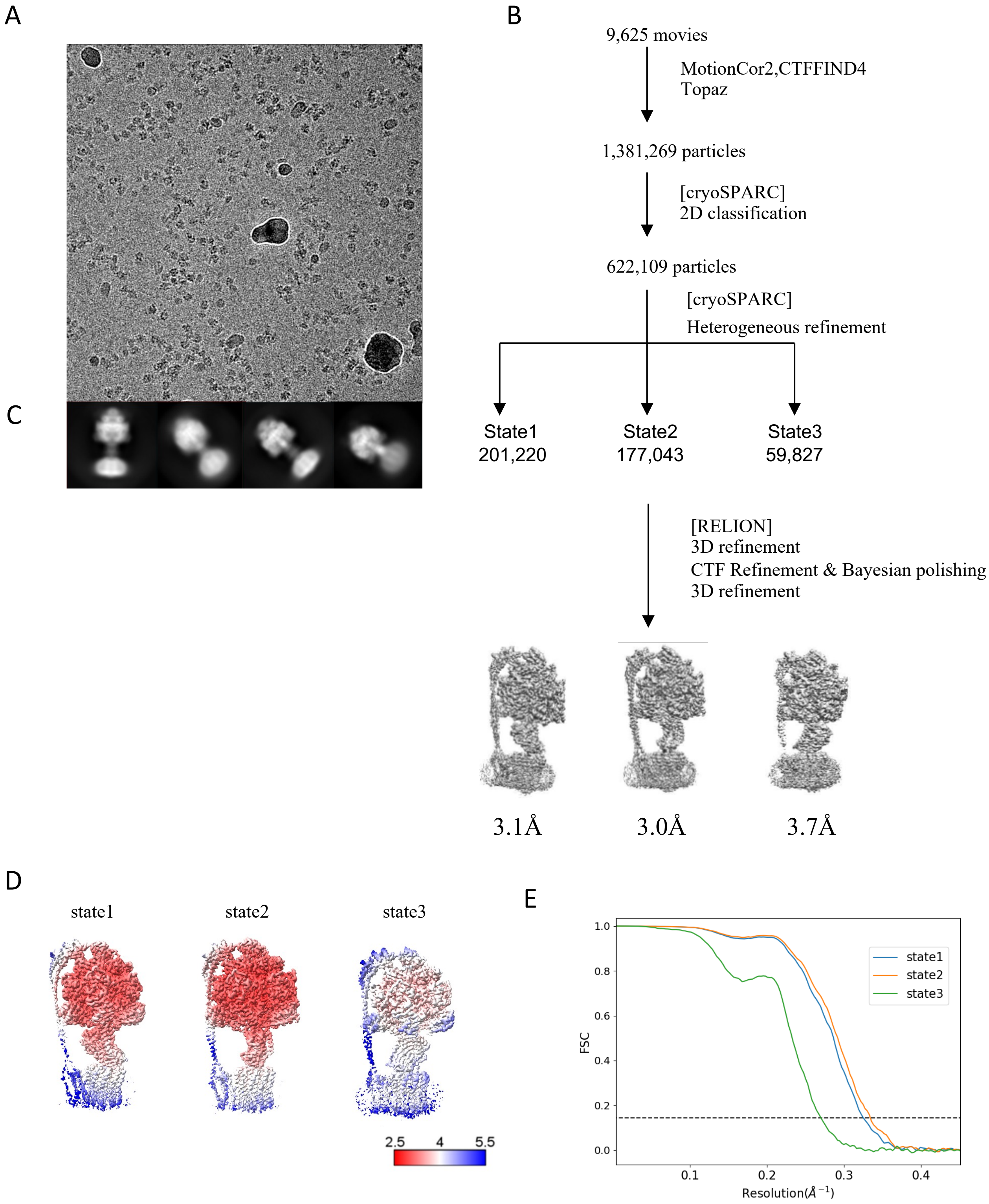

Figure S2. Image acquisition and reconstitution of the 3D structure of nucleotide-depleted (*ND*) wild-type (*wt)*-F_oF_1

(A) Representative motion-corrected and dose-weighted micrographs. **(B)** Workflow for cryo-electron microscopy (cryo-EM) single particle analysis of *ND*-*wt*-F_oF_1. **(C)** 2D class-averaged images. **(D)** Cryo-EM density map. The colors used to represent local resolution are given in the key. **(E)** Fourier shell correlation (FSC) curves for the three rotational states of *wt*-*ND*-F_oF_1 using FSC = 0.143 resolution criterion.

Fig. S3

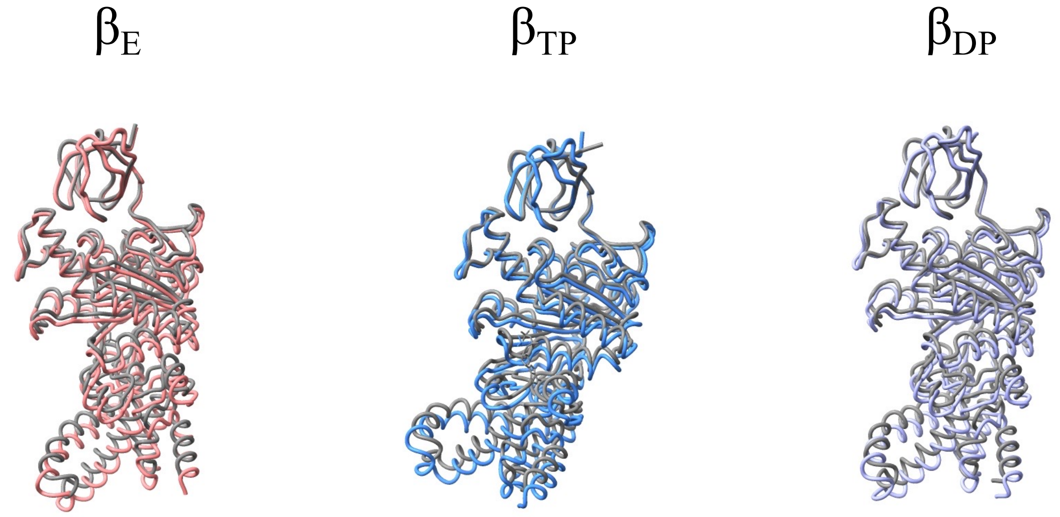

Figure S3. Structure comparison of *wt*-F_oF_1(gray) and *ND-wt*-F_oF_1 (colored). The β_{TP}, β_{DP}, and β_E of *wt*-F_oF_1(6N2Y) and *US-ΔεCT*- F_oF_1 were superimposed in the β -barrel domain (1-80 a .a.), respectively.

Fig. S4

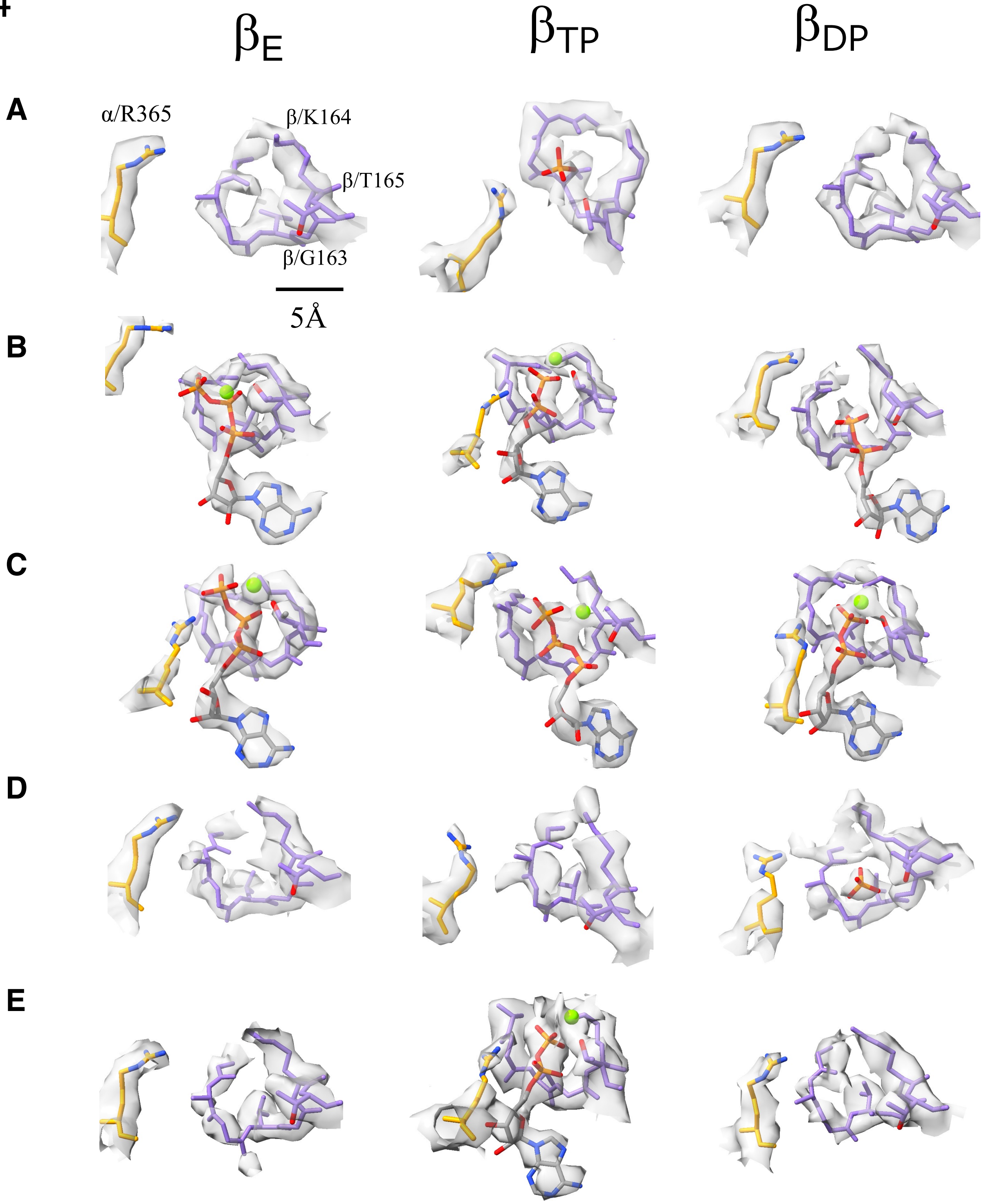

Figure S4. Structure of catalytic sites of wild-type F_oF_1

Magnified views of the three catalytic sites (β_E, β_{TP}, and β_{DP}) in each structure are shown as follows: **(A)** *ND-wt*-F_oF_1, **(B)** *wt*-F_oF_1 ε up form with ATPγS, **(C)** *wt*-F_oF_1 ε down form with ATPγS, **(D)** *ND-ΔCT*-F_oF_1, and **(E)** *US-ΔCT*-F_oF_1. Cryo-electron microscopy maps are represented as semi-translucent structures. Scale bar is 5 Å.

Fig. S5

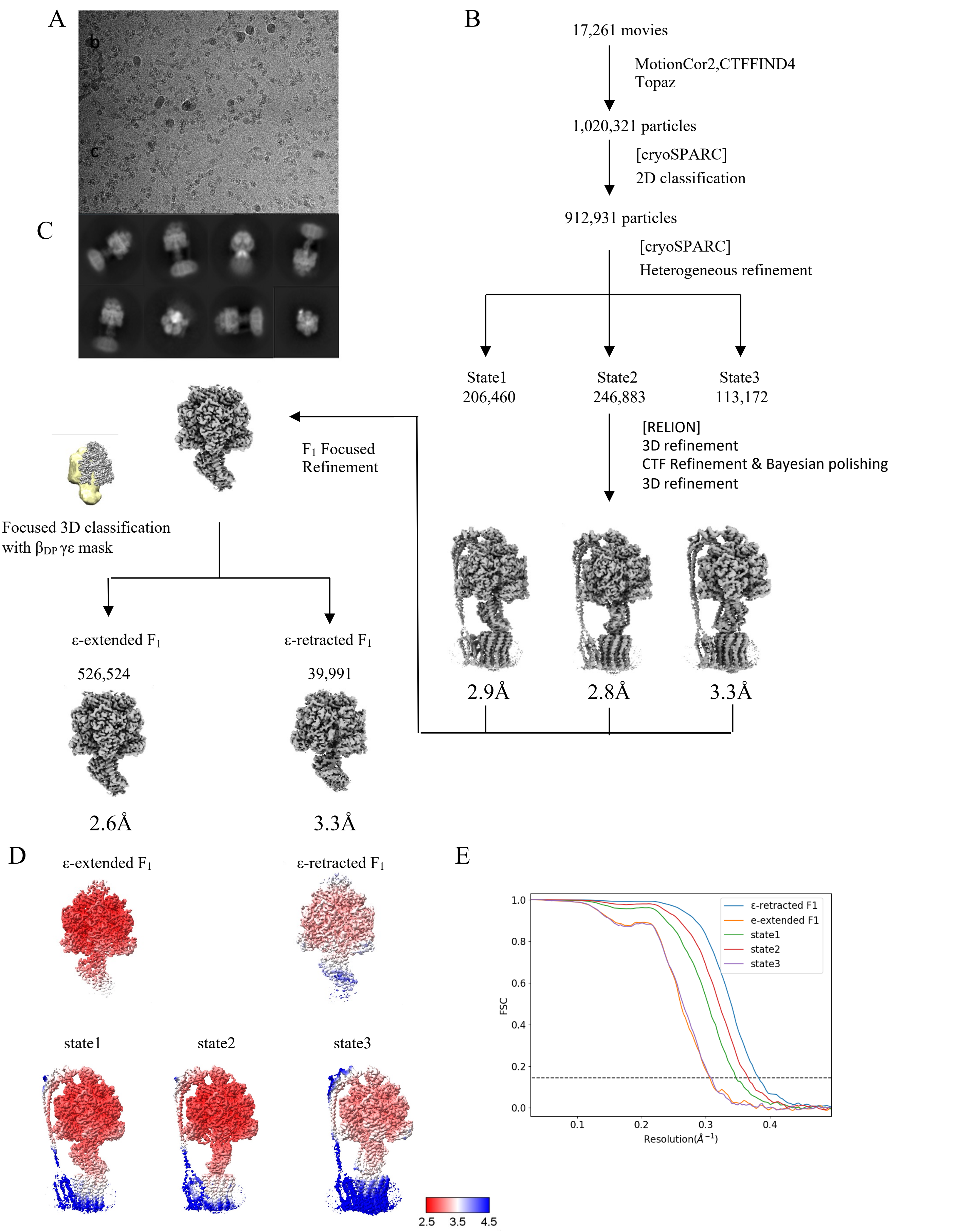
A
B
17,261 movies
MotionCor2,CTFFIND4
Topaz
1,020,321 particles
[cryoSPARC]
2D classification
912,931 particles
[cryoSPARC]
Heterogeneous refinement
State1
206,460
State2
246,883
State3
113,172
[RELION]
3D refinement
CTF Refinement & Bayesian polishing
3D refinement
2.9Å
2.8Å
3.3Å
C
F1 Focused Refinement
Focused 3D classification with βDP γε mask
ε-extended F1
526,524
2.6Å
ε-retracted F1
39,991
3.3Å
D
ε-extended F1
ε-retracted F1
state1
state2
state3
2.5
3.5
4.5
E
ε-retracted F1
e-extended F1
state1
state2
state3
FSC
Resolution(Å−1)
1.0
0.8
0.6
0.4
0.2
0.0
0.1
0.2
0.3
0.4

Figure S5. Image acquisition and reconstitution of the 3D structure of *wt*-F_oF_1 under ATPγS saturation condition

(A) Micrographs. **(B)** Workflow for cryo-electron microscopy (cryo-EM) single particle analysis under ATPγS saturation condition. EM density maps of three states were combined and the F_1 domain was classified into ε-extended and ε-retracted forms. **(C)** 2D class-averaged images. **(D)** Cryo-EM density map of ε-extended and ε-retracted F_1 domains. The colors used to represent local resolution are given in the key. **(E)** Fourier shell correlation (FSC) curves for ε-extended and ε-retracted F_1 domains using FSC = 0.143 resolution criterion.

Fig. S6

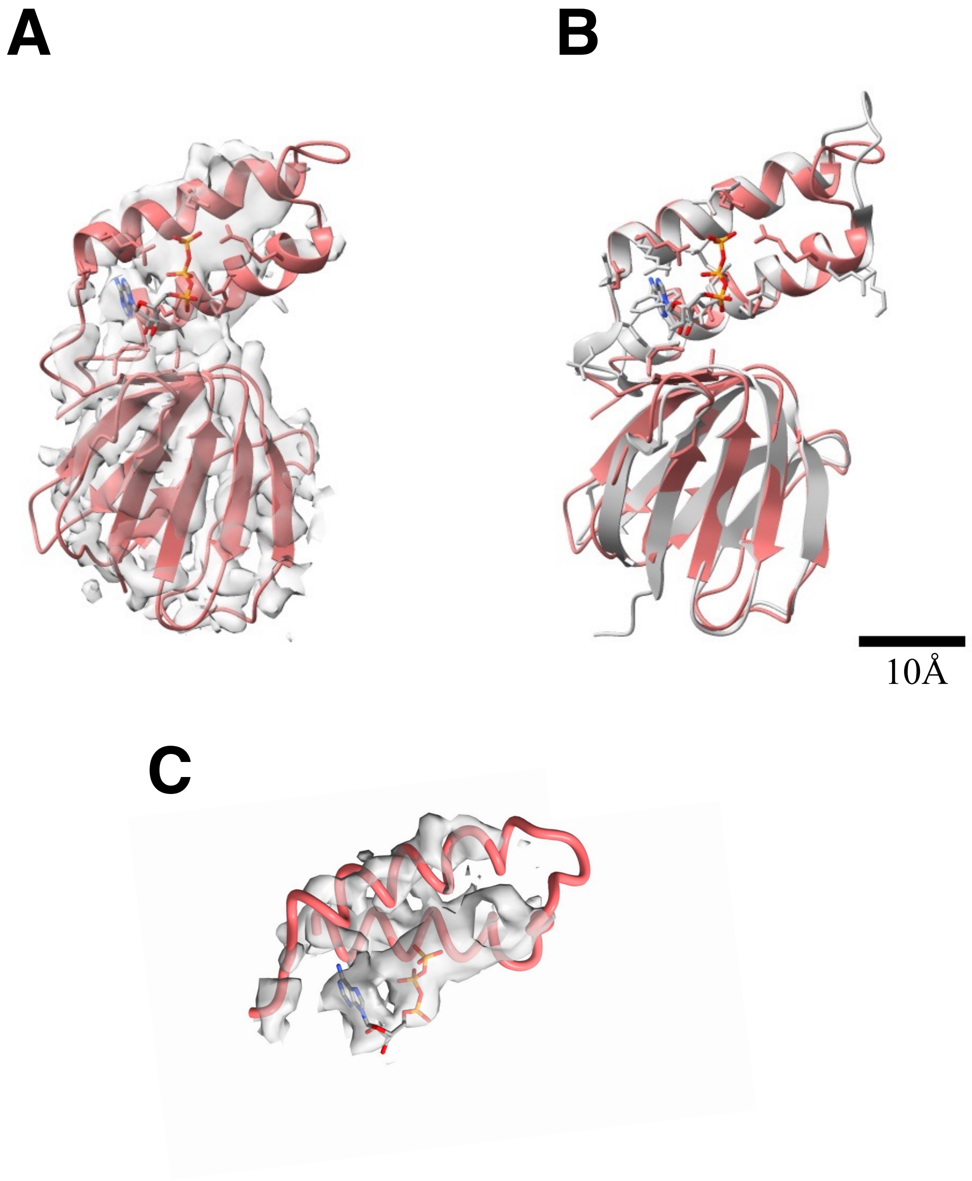

Figure S6. Structure of down form of ε subunit with ATP in *wt*-F_oF_1
(A) Density map of ε subunit in F_1 domain obtained under saturated ATPγS condition. (**B**) Superposition of the crystal structure of retracted-ε subunit (gray, PDB:2E5Y) and the structure of retracted-ε in F_1 domain obtained under saturated ATPγS condition. (**C**) Magnified view of ATP binding site of retracted-ε in F_1 domain. ATP was represented as sticks.

Fig. S7

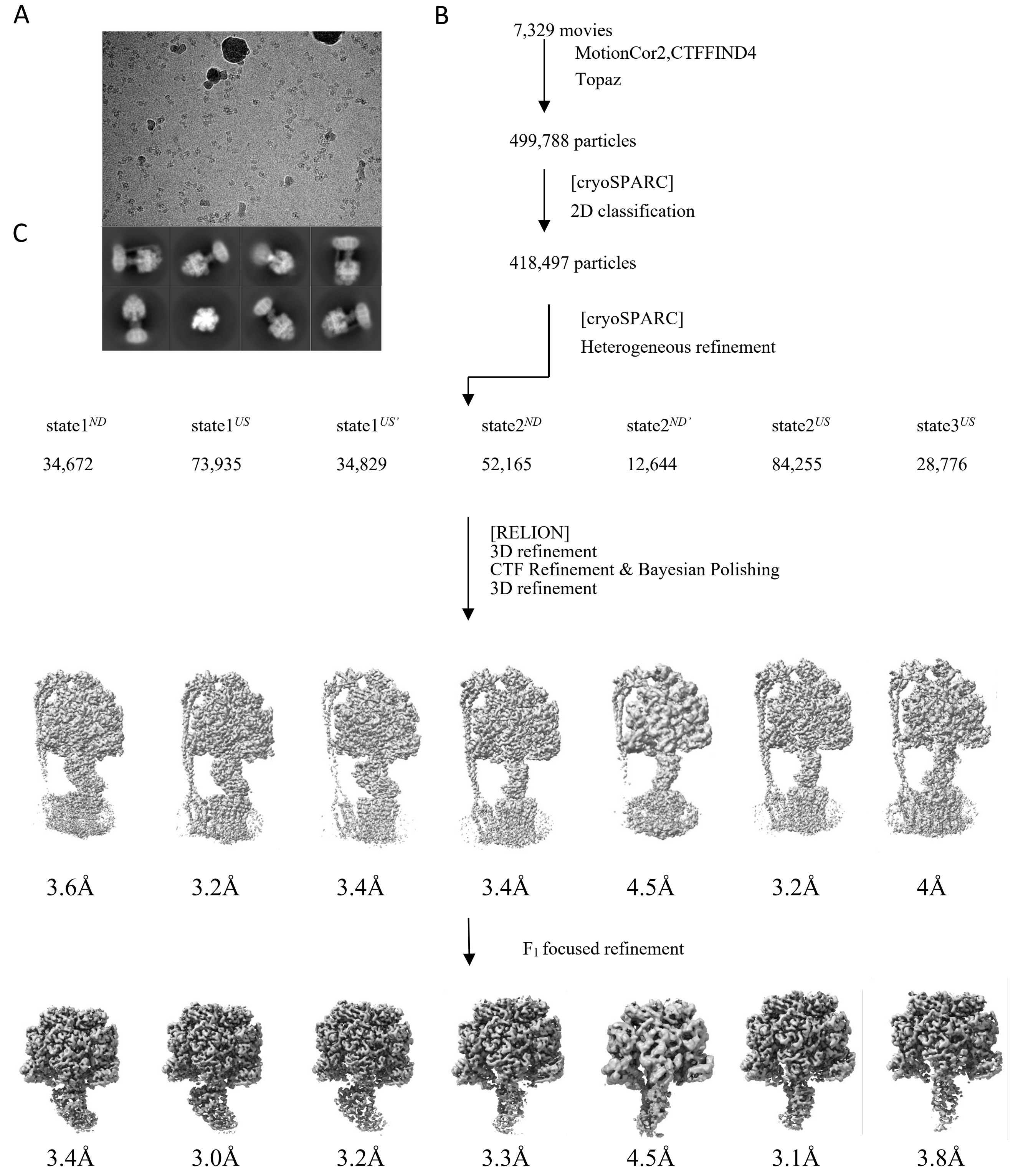
A
B
C
7,329 movies
MotionCor2,CTFFIND4
Topaz
499,788 particles
[cryoSPARC]
2D classification
418,497 particles
[cryoSPARC]
Heterogeneous refinement
state1ND
state1US
state1US'
state2ND
state2ND'
state2US
state3US
34,672
73,935
34,829
52,165
12,644
84,255
28,776
[RELION]
3D refinement
CTF Refinement & Bayesian Polishing
3D refinement
3.6Å
3.2Å
3.4Å
3.4Å
4.5Å
3.2Å
4Å
F1 focused refinement
3.4Å
3.0Å
3.2Å
3.3Å
4.5Å
3.1Å
3.8Å

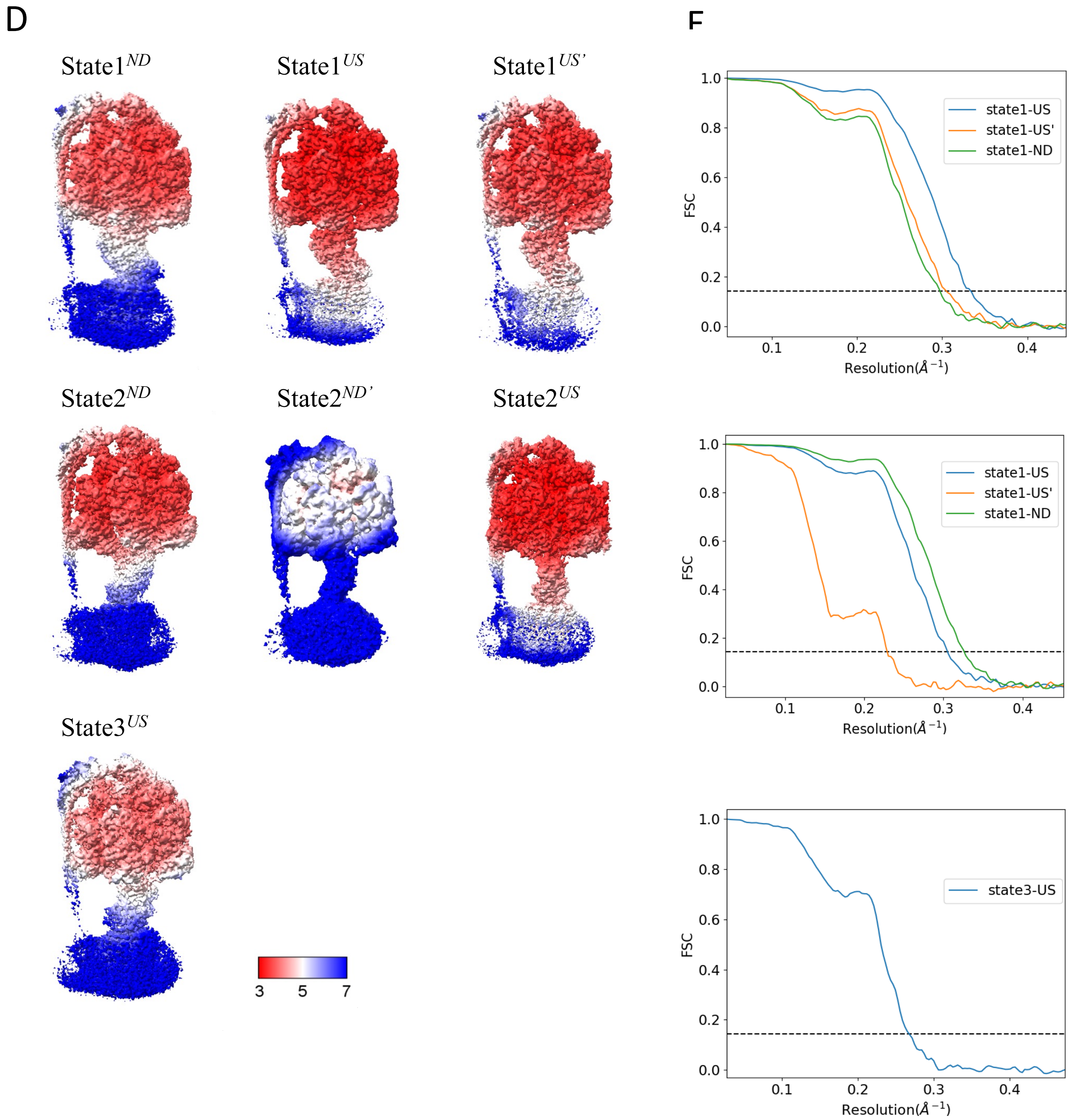

Figure S7. Image acquisition and reconstitution of the 3D structures of nucleotide-depleted (*ND*)-*ΔεCT*-F_oF_1 and Uni-Site (*US*)-*ΔεCT*-F_oF_1.

(A) Micrographs. **(B)** Workflow for cryo-electron microscopy (cryo-EM) single particle analysis under unisite conditions. **(C)** 2D class-averaged images. **(D)** Cryo-EM density map of *ND-ΔεCT*-F_oF_1 and *US-ΔεCT*-F_oF_1. The colors used to represent local resolution are given in the key. **(E)** Fourier shell correlation (FSC) curves for each of the three rotational states of F_oF_1 obtained from the unisite condition using FSC = 0.143 for resolution criterion.

Fig. S8

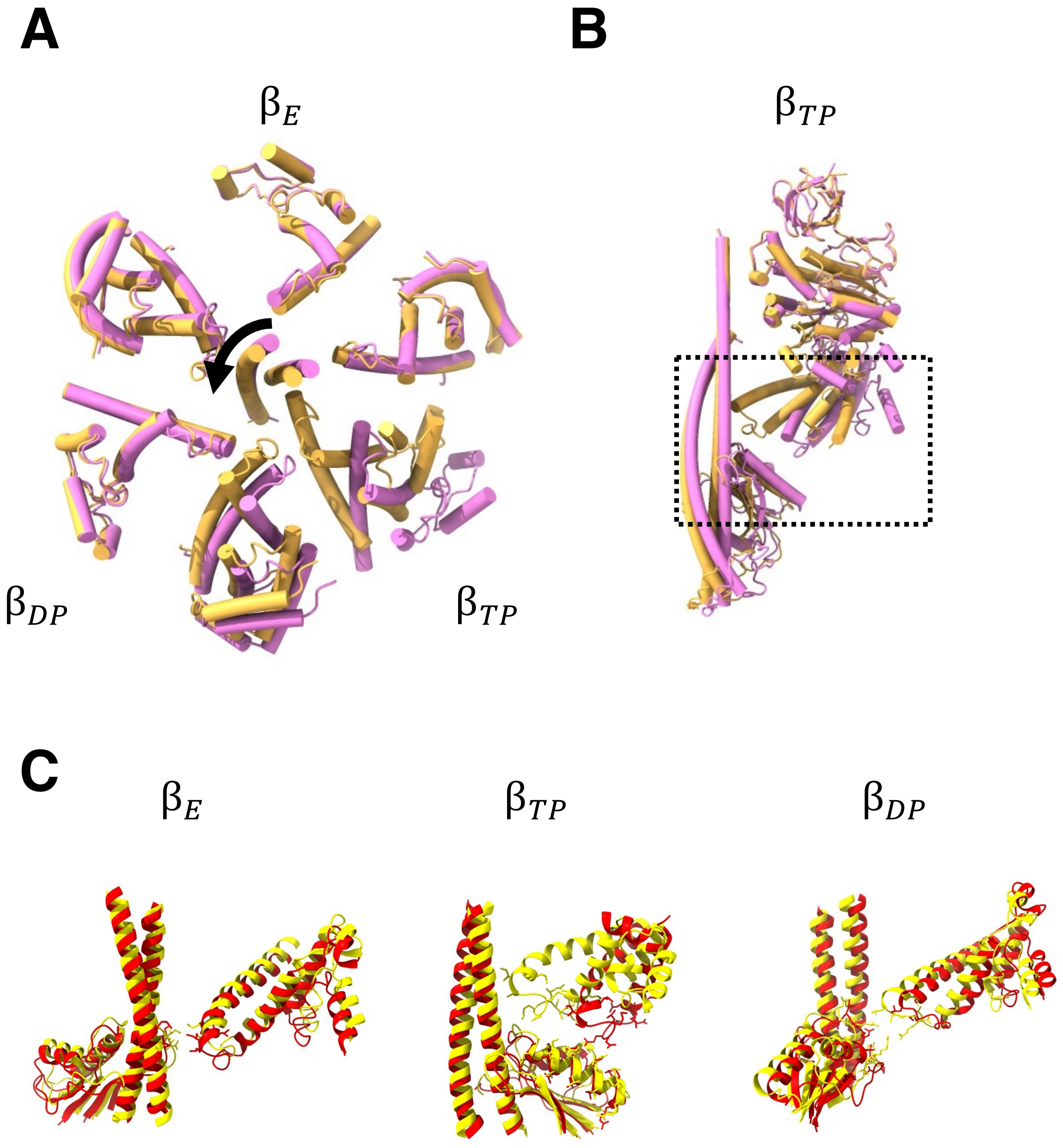

Figure S8. Structure comparison of *US-ΔεCT*-F_oF_1 (orange) and *ND-wt*-F_oF_1(pink). (**A**) slice view of C terminal F_1 domain. (**B**) Side view of β_{TP} and γ subunit. All subunits were represented as cylinders.

Fig. S9

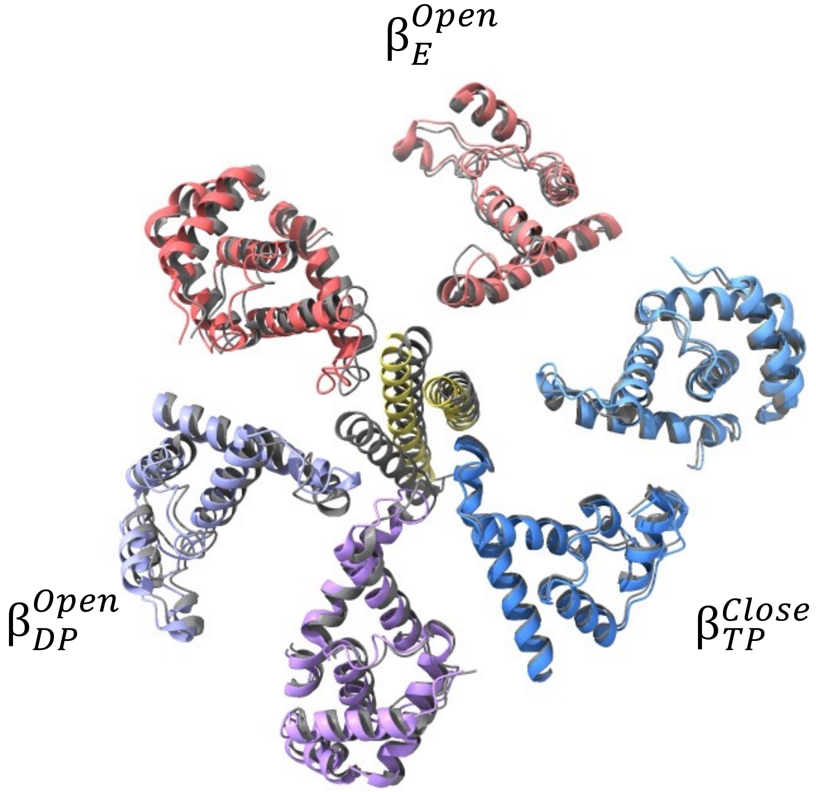

Figure S9. Structure comparison of *US-ΔεCT*-F_oF_1 (colored) and *ND-wt*-F_oF_1(gray) in C termini domain of both α and β subunits, and γ subunit.

Fig. S10

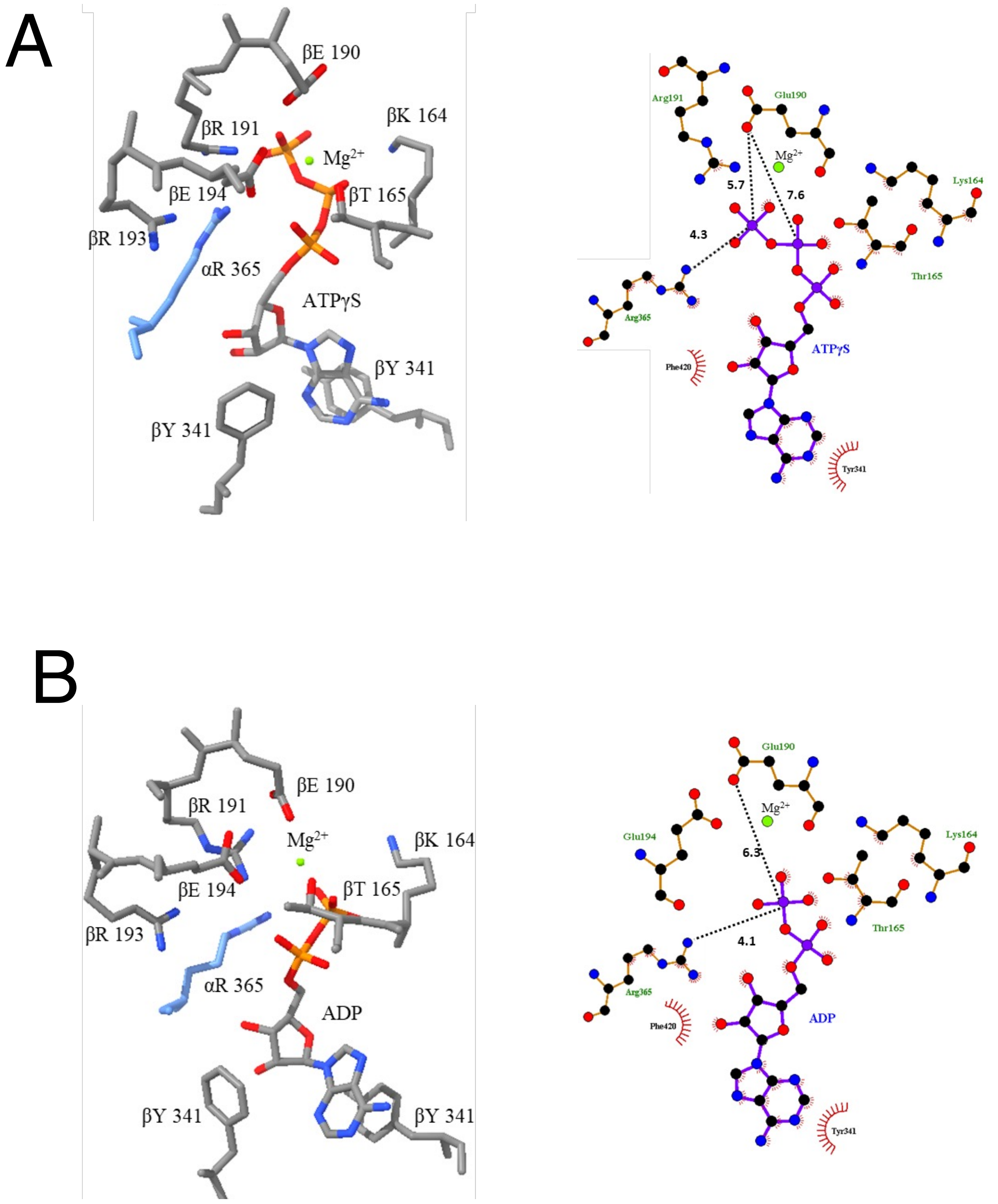

Figure S10. Coordination of nucleotides in the binding sites of wt-F_oF_1 ε-retracted form with ATPγS. *Left panels;* binding sites of β_{TP} (**A**) and β_{DP} (**B**) in *wt*-F_oF_1 ε-retracted form with ATPγS (drawn with colored atoms and bonds and blue main chain) *Right panels;* Schematic representations of the coordination of ATP group bound to the side chains, respectively. The distances between the atoms are shown in dotted lines. All distances are in Å. Two-dimensional diagram generated by Ligplot.

Supplementary Table1. ATPase activity of *wt*-F_oF_1 and *ΔεCT* -F_oF_1 at the indicated time after initiation of reaction. The activities of wild-type F_oF_1 and *ΔεCT*-F_oF_1 were measured using the enzyme coupling assay described in the Materials and Methods section.

time	30 s	600 s	1000 s
wt-F_oF_1	11 s^{-1}	41 s^{-1}	51 s^{-1}
ΔεCT-F_oF_1	49 s^{-1}	107 s^{-1}	130 s^{-1}

Supplementary Table 2. Cryo-EM data collection, refinement and validation statistics for *wt-ND* F_oF_1.

	state1 F_oF_1	state2 F_oF_1	state3 F_oF_1
EMDB ID	33251	33252	33253
PDB ID	7XKH		
**Data collection and processing**			
Magnification	50,000	50,000	50,000
Microscope	CRYOARM300	CRYOARM300	CRYOARM300
Voltage(kV)	300	300	300
Total dose (e^-/ Å^2)	50	50	50
Pixel size(Å /pix)	1.01	1.01	1.01
Defocus range(μm)	-0.8 to -2.0	-0.8 to -2.0	-0.8 to -2.0
symmetry imposed	C1	C1	C1
Initial particle	1,381,269	1,381,269	1,381,269
Final Particle	201,220	177,043	59,827
Map resolution(Å)	3.1	3	3.7
FSC threshold	0.143	0.143	0.143
**Refinement**			
Initial model used	This study	This study	This study
Model resolution	3.1		
FSC threshold	0.5	0.5	0.5
**Model composition**			
Nonhydrogen atoms	25,563		
Protein residues	3,317		
Ligands	Pi		
**R.m.s deviations**			
Bond length (Å)	0.002		
Bond Angles (°)	0.443		
**Validation**			
MolProbity score	1.04		
EMRinger score	2.56		
Clashscore	2.56		
Rotamer outlier (%)	0		
CaBALM outlier (%)	1.67		
**Ramachandran plot**			
Favored (%)	98.36		
Allowed (%)	1.64		
Disallowed (%)	0		

Supplementary Table 3. Cryo-EM data collection, refinement and validation statistics for *wt*-F_oF_1 obtained under 4 mM ATPγS condition.

	ε-extended F_1 domain	ε-retracted F_1 domain	state1 F_oF_1	state2 F_oF_1	state3 F_oF_1
EMDB ID	33264	33265	33266	33267	33268
PDB ID	7XKQ	7XKR			
**Data collection and processing**					
Magnification	50,000	81,000	81,000	81,000	81,000
Microscope	Titan Krios	Titan Krios	Titan Krios	Titan Krios	Titan Krios
Voltage(kV)	300	300	300	300	300
Total dose (e^-/Å^2)	50	50	50	50	50
Pixel size(Å /pix)	0.88	0.88	0.88	0.88	0.88
Defocus range(μm)	-0.8 to -2.0	-0.8 to -2.0	-0.8 to -2.0	-0.8 to -2.0	-0.8 to -2.0
symmetry imposed	C1	C1	C1	C1	C1
Initial particle	1,020,321	1,020,321	1,020,321	1,020,321	1,020,321
Final Particle	526,524	39,991	206,460	246,883	113,172
Map resolution(Å)	2.6	3.3	2.9	2.8	3.3
FSC threshold	0.143	0.143	0.143	0.143	0.143
**Refinement**					
Initial model used	This study	This study	This study	This study	This study
Model resolution	2.7	3.5			
FSC threshold	0.5	0.5	0.5	0.5	0.5
**Model composition**					
Nonhydrogen atoms	25,254	25,306			
Protein residues	3,257	3,259			
Ligands	5MG, 4ATP, 2ADP	6MG, 6ATP, ADP			
**R.m.s deviations**					
Bond length (Å)	0.003	0.003			
Bond Angles (°)	0.545	0.507			
**Validation**					
MolProbity score	1.1	1.27			
EMRinger score	4.29	3.31			
Clashscore	3.13	4.92			
Rotamer outlier (%)	0	0			
CaBALM outlier (%)	1.3	1.85			
**Ramachandran plot**					
Favored (%)	98.36	97.96			
Allowed (%)	1.64	2.01			
Disallowed (%)	0	0			

Supplementary Table 4. Cryo-EM data collection, refinement and validation statistics for *ΔCT*-F_oF_1 obtained under unisite.

	state1 ND		state1 US		state1 US'	
	F_oF_1	F_1	F_oF_1	F_1	F_oF_1	F_1
EMDB ID	33277	33258	33278	33259	33279	33260
PDB ID		7XKO		7XKP		
**Data collection and processing**						
Magnification	81,000	81,000	81,000	81,000	81,000	81,000
Microscope	Titan Krios	Titan Krios	Titan Krios	Titan Krios	Titan Krios	Titan Krios
Voltage(kV)	300	300	300	300	300	300
Total dose (e^-/Å^2)	60	60	60	60	60	60
Pixel size(Å/pix)	0.88	0.88	0.88	0.88	0.88	0.88
Defocus range(μm)	-0.8 to -2.0	-0.8 to -2.0	-0.8 to -2.0	-0.8 to -2.0	-0.8 to -2.0	-0.8 to -2.0
symmetry imposed	C1	C1	C1	C1	C1	C1
Initial particle	499,788	499,788	499,788	499,788	499,788	499,788
Final Particle	34,672	34,672	73,935	73,935	34,829	34,829
Map resolution(Å)	3.6	3.4	3.2	3	3.4	3.2
FSC threshold	0.143	0.143	0.143	0.143	0.143	0.143
**Refinement**						
Initial model used	This study	This study	This study	This study	This study	This study
Model resolution		3.5				
FSC threshold	0.5	0.5	0.5	0.5	0.5	0.5
**Model composition**						
Nonhydrogen atoms		24,070		24,594		
Protein residues		3,127		3,188		
Ligands		Pi		ATP, MG		
**R.m.s deviations**						
Bond length (Å)		0.003		0.002		
Bond Angles (°)		0.49		0.48		
**Validation**						
MolProbity score		1.47		1.25		
EMRinger score		2.61		3.37		
Clashscore		5.68		3.86		
Rotamer outlier (%)		0		0		
CaBALM outlier (%)		1.9		1.87		
**Ramachandran plot**						
Favored (%)		97.08		97.67		
Allowed (%)		2.92		2.33		
Disallowed (%)		0		0		

Supplementary Table 5. Cryo-EM data collection, refinement and validation statistics *ΔCT*-F_oF_1 obtained under unisite condition.

	state2 ND		state2 ND'		state2 US		state3 US	
	F_oF_1	F_1	F_oF_1	F_1	F_oF_1	F_1	F_oF_1	F_1
EMDB ID	33280	33261	33281	33269	33282	33262	33283	33263
PDB ID								
**Data collection and processing**								
Magnification	81,000	81,000	81,000	81,000	81,000	81,000	81,000	81,000
Microscope	Titan Krios	Titan Krios	Titan Krios	Titan Krios	Titan Krios	Titan Krios	Titan Krios	Titan Krios
Voltage(kV)	300	300	300	300	300	300	300	300
Total dose (e^-/Å ^{2})	60	60	60	60	60	60	60	60
Pixel size(Å /pix)	0.88	0.88	0.88	0.88	0.88	0.88	0.88	0.88
Defocus range(μm)	-0.8 to -2.0	-0.8 to -2.0	-0.8 to -2.0	-0.8 to -2.0	-0.8 to -2.0	-0.8 to -2.0	-0.8 to -2.0	-0.8 to -2.0
symmetry imposed	C1	C1	C1	C1	C1	C1	C1	C1
Initial particle	499,788	499,788	499,788	499,788	499,788	499,788	499,788	499,788
Final Particle	52,165	52,165	12,644	12,644	84,255	84,255	28,776	28,776
Map resolution(Å)	3.4	3.3	4.5	4.5	3.2	3.1	4	3.8
FSC threshold	0.143	0.143	0.143	0.143	0.143	0.143	0.143	0.143
